# Is singlet oxygen involved in FLASH‐RT?

**DOI:** 10.1002/acm2.13974

**Published:** 2023-03-25

**Authors:** Ahmed Alanazi, Jean‐Paul Jay‐Gerin, Alfonso Blázquez‐Castro

**Affiliations:** ^1^ Département de Médecine Nucléaire et de Radiobiologie Faculté de Médecine et des Sciences de la Santé, Université de Sherbrooke Sherbrooke QC Canada; ^2^ Departamento de Biología Universidad Autónoma de Madrid Madrid Spain

Advances in modalities and radiation delivery systems in the field of radiation therapy (RT) have led to concentrated efforts on how to improve the protection of normal tissue and avoid adverse effects, the most critical element in radiotherapy. In 2014, a new irradiation method called “ultra‐high dose rate radiotherapy” or “FLASH‐RT^1^” demonstrated a sparing effect of surrounding normal tissues without compromising the anti‐tumor action. Although several hypotheses have been proposed to explain this phenomenon (for a review, see, e.g., Ref. 2); however, no consideration has been given to date on the potential role and importance of singlet oxygen (^1^O_2_) in FLASH‐RT. Reactive oxygen species (ROS) play an important role in the regulation of many cellular processes, including cell death and DNA repair. Identifying the differential response to FLASH‐RT in normal and malignant tissue therefore critically depends on a thorough understanding of the mechanisms of ROS formation and action in vivo. Although singlet oxygen is one of the most reactive intermediates in biological systems, its impact has often been overlooked. In this context, it is the purpose of this Letter to show that the study of singlet oxygen yields at ultra‐high dose rates could provide critical insights into our understanding of the FLASH‐RT effect.

Examining the underlying physical processes generated by FLASH‐RT can provide valuable insights into the nature of the biologically significant effects. Clearly, the nature of the dominant primary component generated by FLASH‐RT is qualitatively and quantitatively distinct from that of conventional low radiation dose rates. Different biological responses are observed depending on the intensity and on the way the initial ionizing radiation is delivered. These responses depend, at least in part, on various physicochemical processes that may occur at different thresholds and at different levels depending in particular on the doses and/or dose rates involved. In the case of large doses and/or as a result of ultra‐high dose rates (UHDR), ionizing radiation could generate unique events that could be achieved by some physical phenomena, such as “dissociative electron attachment” and Cherenkov radiation. As a result of such processes and consistent with the FLASH effect, singlet oxygen may be one of the most significant ROS involved in FLASH‐RT. This could thus open a new research avenue aimed at explaining the FLASH effect.

Singlet oxygen can be generated in various ways in a biological system exposed to ionizing radiation. For example, one potential mechanism for the formation of ^1^O_2_ is through “dissociative electron attachment” (DEA), during which a low‐energy (<20 eV) secondary electron attaches to an oxygen molecule in its triplet ground state (^3^O_2_).^3^ Briefly, an electron with a kinetic energy typically of 20 eV or less temporarily attaches to an oxygen molecule to form an electronically excited superoxide anion radical (O_2_ **^•^** ^‐^)*. This anion is unstable and quickly dissociates ejecting the bound extra electron with some energy (lower than its incident initial energy), while leaving the oxygen molecule in an excited state (^1^O_2_) (see Figure 1). This mechanism is inefficient during conventional irradiation at low dose rate. However, it can become very important at high (FLASH) dose rates where a large number of low‐energy secondary electrons are produced throughout the irradiated volume, thus being able to convert very quickly via DEA (viz, on a picosecond time scale) most of the oxygen present in this volume into singlet oxygen. This can thus be considered as an “oxygen depletion” effect in the sense that ^1^O_2_ is not known to be a radiosensitizer like ^3^O_2_. In other words, this mechanism would cause radioprotection in normal cells when exposed to high dose‐rate irradiation^4^ due to their lower ROS susceptibility as compared to tumor cells (see *infra*).

**FIGURE 1:**
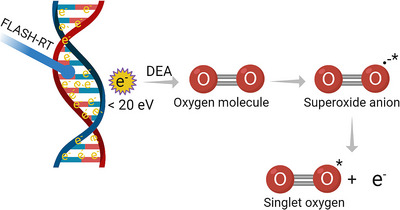
Illustration of the dissociative electron attachment (DEA) process in FLASH‐RT and presumable singlet oxygen production pathway.

Singlet oxygen has been investigated in a large number of studies regarding its formation, reactions, and biological properties_._ It is involved in a number of redox processes due to its unique chemical nature and strong oxidizing properties.^5^ However, the oxidation process involving singlet oxygen could be affected by a number of different circumstances, including: (1) the site of ^1^O_2_ generation, which must be close to the target to cause high levels of oxidative damage, as shown, for example, in nuclear DNA^6^; (2) the selectivity of ^1^O_2_ with a variety of biomolecules,^7^ which allows it to play an important role in a number of biological processes; (3) ^1^O_2_ can be efficiently quenched by many compounds, either by chemical scavenging (“chemical quenching”) or “physical quenching” in which the electronic excitation energy is rapidly degraded, then yielding the ^3^O_2_ ground state and waste heat^7^; and most importantly; and (4) ^1^O_2_ can contribute significantly to the production of the highly mutagenic oxidized base 8‐oxo‐7,8‐dihydroguanine in DNA.^6^ However, in primary non‐transformed mammalian cells, the extensive DNA oxidation induced by 8‐oxo‐7,8‐dihydroguanine can be rapidly and efficiently repaired by DNA repair mechanisms, thereby maintaining biological functions.^8^, ^9^

The particular mechanism(s) responsible for FLASH‐RT sparing of normal tissues still remains elusive today, despite many efforts devoted to a better understanding of the FLASH effect. It is known that tumor cells, in general, have a more pro‐oxidant intracellular redox state than normal cells.^10^, ^11^ This is because of a higher ROS concentration and/or a lower antioxidant activity in the cancer cells. As a consequence, tumor cells are closer to reaching a non‐return cell death threshold when exposed to a ROS surge. This has been shown experimentally repeatedly.^12^, ^13^ In fact, because of this phenomenon, radiotherapy‐ or chemotherapy‐driven ROS overload is being proposed as an effective approach to promote cancer cell death while inducing much less damage to normal tissues.^14^, ^15^

In conclusion, it is our opinion that future studies should place more emphasis on the role and importance of physical events related to the properties of radiation. In this context, the possibility of the intervention of singlet oxygen in the mechanism(s) underlying FLASH‐RT should be considered. Validation of such a hypothesis would require studies on the yields of singlet oxygen generated by FLASH‐RT in living tissues.

## CONFLICT OF INTEREST STATEMENT

The authors have no conflicts of interest to declare.

## CONSENT FOR PUBLICATION

The authors agree with the paper's content.
